# *eNOS* polymorphisms on male infertility: An updated systematic review and meta-analysis

**DOI:** 10.1097/MD.0000000000033993

**Published:** 2023-06-16

**Authors:** Zhihai Teng, Hu Wang, Fengran Guo, Zhenwei Han, Yaxuan Wang

**Affiliations:** a Department of Urology, The Second Hospital of Hebei Medical University, Shijiazhuang, China.

**Keywords:** eNOS, male infertility, meta-analysis, polymorphism, SNP

## Abstract

This meta-analysis was performed to examine the association of 3 endothelial nitric oxide synthase (eNOS) gene polymorphisms with male infertility.

**Methods:**

The literature on the relation between the mutant of eNOS and male infertility before July 1, 2022, was conducted in Pubmed, Medline, and Web of Science. The search strategy is as follows: (eNOS OR ECNOS OR nitric oxide synthase 3 OR NOS3) AND (polymorphism OR mutation OR variation OR SNP OR genotype) AND (male infertility). Statistical analysis was performed with the web of MetaGenyo, Stata 12, trial sequential analysis 0.9Beta, and the web of GTEx.

**Results:**

Overall, 13 studies (26 case-controls) were included involving 6518 cases and 5461 controls for 3 polymorphisms (rs2070744, rs1799983, rs61722009) of eNOS. We found that eNOS rs2070744 was correlated with an increased risk of male infertility (C vs. T: odds ratio [OR], 1.48; 95% confidence interval [CI], [1.19–1.85]; CC vs. TT: OR, 2.59; 95% CI, [1.40–4.80]; CT vs. TT: OR, 1.17; 95% CI, [1.00–1.38]; CC vs. CT + TT: OR, 2.50; 95% CI, [1.35–4.62]; CC + CT vs. TT: OR, 1.41; 95% CI, [1.21–1.64]). And eNOS rs1799983 was correlated with an increased risk of male infertility (allele contrast T vs. G: OR, 1.41; 95% CI, [1.01–1.96]; *P* = .043; recessive model TT vs. TG + GG: OR, 2.00; 95% CI, [1.03–3.90]; *P* = .042). In the stratified analysis of rs61722009, we found Asians might be correlated with an increased risk of male infertility (4a vs. 4b: OR, 1.50; 95% CI, [0.94–2.38]; 4a4a vs. 4b4b: OR, 2.56; 95% CI, [0.70–9.38]; 4a4b vs. 4b4b: OR, 1.36; 95% CI, [0.87–2.13]; 4a4a vs. 4a4b + 4b4b: OR, 2.57; 95% CI, [0.91–7.30]; 4a4a + 4a4b vs. 4b4b: OR, 1.44; 95% CI, [0.87–2.40]).

**Conclusion:**

The eNOS rs2070744 polymorphism and rs1799983 are associated with the risk of male infertility, and rs61722009 might be a risk factor for Asians.

## 1. Introduction

Male infertility has significantly impacted 10 to 15% of the world’s couples and has gradually become a common disease.^[1]^ The trouble in the relations and the community is raised. There are several factors for male infertility, including environmental changes and genetic mutations,^[2,3]^ among others. And these factors have a negative impact on sperm function.^[4]^ Previous studies have identified three types of male infertility including azoospermia, asthenozoospermia, and oligozoospermia.

Oxidative stress is a significant problem linked to sperm dysfunction, affecting sperm motility and asthenozoospermia.^[5]^ Nitrogen oxide (NO) is a reactive free radical gas with diverse functions, acting as a messenger in numerous biological processes.^[6]^ Previously studies have demonstrated that NO is synthesized by human male gametes and plays a role in mammalian reproduction and sexual function.^[7,8]^ However, excessive concentrations of NO can lead to dysfunction, while low concentrations of NO play a crucial role in spermatozoon physiology.^[9]^

NO is mainly produced by nitric oxide synthase (NOS) by the conversion of L-arginine to L-citrulline.^[10]^ NOS consists of 3 isoforms: endothelial NOS (eNOS), inducible NOS, and neuronal NOS.^[11]^ The eNOS is the main enzyme to produce NO. The eNOS gene contains 3 common single nucleotide polymorphisms known as rs2070744, rs1799983, and rs6172009. Studies have shown that these eNOS polymorphisms are associated with oxidative stress in various clinical conditions, including pre-eclampsia, persistent obstructive pulmonary disorders, diabetic nephropathy, and others.^[12,13]^

In recent years, several randomized controlled trials have been conducted to investigate the association between male infertility and the polymorphisms of eNOS, and a few meta-analyses were used to verify the results. Due to limitations in sample sizes, the findings of these studies have been inconsistent. So we provided a comprehensive review to examine the associations between them.

## 2. Methods

### 2.1. Literature search

We conducted a comprehensive literature search on PubMed, Medline, and Web of Science up to July 1, 2022, to investigate the potential association between eNOS polymorphisms and male infertility risk. Our search strategy included the terms “eNOS,” “ECNOS,” “nitric oxide synthase 3,” “NOS3,” “polymorphism,” “mutation,” “variation,” “SNP,” “genotype,” and “male infertility,” and we only included studies published in English. Following a meticulous screening process, we identified 3 eNOS polymorphisms (rs2070744, rs1799983, and rs6172009) for further investigation. We conducted a comprehensive review of the selected studies, ensuring their high quality and relevance to our research question.

### 2.2. Inclusion criteria and exclusion criteria

Our meta-analysis followed a set of inclusion and exclusion criteria for selecting articles. We included case-control studies that investigated the association between eNOS polymorphisms and cancer risk, as well as population genetic polymorphism publications. Additionally, we incorporated articles that provided sufficient genotype data, allowing us to estimate odds ratios (ORs) and corresponding 95% confidence intervals (CIs). We also required that control subjects satisfied Hardy–Weinberg equilibrium (HWE). We excluded case-only studies, case reports, or reviews, studies without raw data for eNOS genotypes, and studies that combined other influencing factors. By applying these criteria, our aim is to ensure that the studies included in our meta-analysis were of high quality and directly relevant to our research objectives.

### 2.3. Data extraction

Two researchers obtained the data independently, following the selection criteria outlined above, and any discrepancies were resolved through consensus. The data extracted from the eligible articles included the first author’s name, publication year, ethnicity, source of control, and the number of cases and controls with eNOS genotypes. Ethnicity was categorized into 3 groups: “Mixed,” “Caucasian,” and “Asian.” These data were carefully collected and will be used in our subsequent analysis to assess the potential association between eNOS polymorphisms and disease risk. Our rigorous data collection process ensures the reliability and accuracy of our meta-analysis

### 2.4. Statistical analysis

Aggregate ORs and corresponding 95% CIs were calculated to assess the association between eNOS polymorphism and cancer risk, using allelic (B vs. A), homozygote (BB vs. AA), heterozygote (BA vs. AA), dominant (BA + BB vs. AA), and recessive (BB vs. BA + AA) models (A = wild allele; B = mutated allele). The heterogeneity between studies was evaluated using the Cochrane *Q*-statistic test, and the unreliability was quantified using the *I*^2^ statistic. A random effects model was used if *I*^2^ > 50% or P_Q_ ≤ 0.1, indicating substantial heterogeneity, otherwise, a fixed effects model was applied. Subgroup meta-analysis was performed by ethnicity and the source of control. Sensitivity analysis was also conducted by omitting 1 study each time to assess the stability of the results. The HWE was estimated using the asymptotic test, and deviation was considered when *P* < .05. The potential publication bias of the eligible studies was quantitatively evaluated using the Egger regression test. Statistical analysis was performed using Stata 12.0 software (version 12.0; State Corporation, College Station, TX) and the web of MetaGenyo (https://metagenyo.genyo.es/).^[14]^ A 2-tailed *P* < .05 was considered statistically significant.

### 2.5. In-silico analysis using GTEx website

For the purpose of investigating the influence of polymorphisms on eNOS, we obtained the association between the polymorphism and eNOS expression level using the GTEx cohort.^[15]^

### 2.6. Trial sequential analysis (TSA)

TSA was performed to reduce random errors and strengthen the robustness of our decisions.^[16]^

## 3. Results

### 3.1. Main characteristics of the enrolled studies

**Figure 1.**
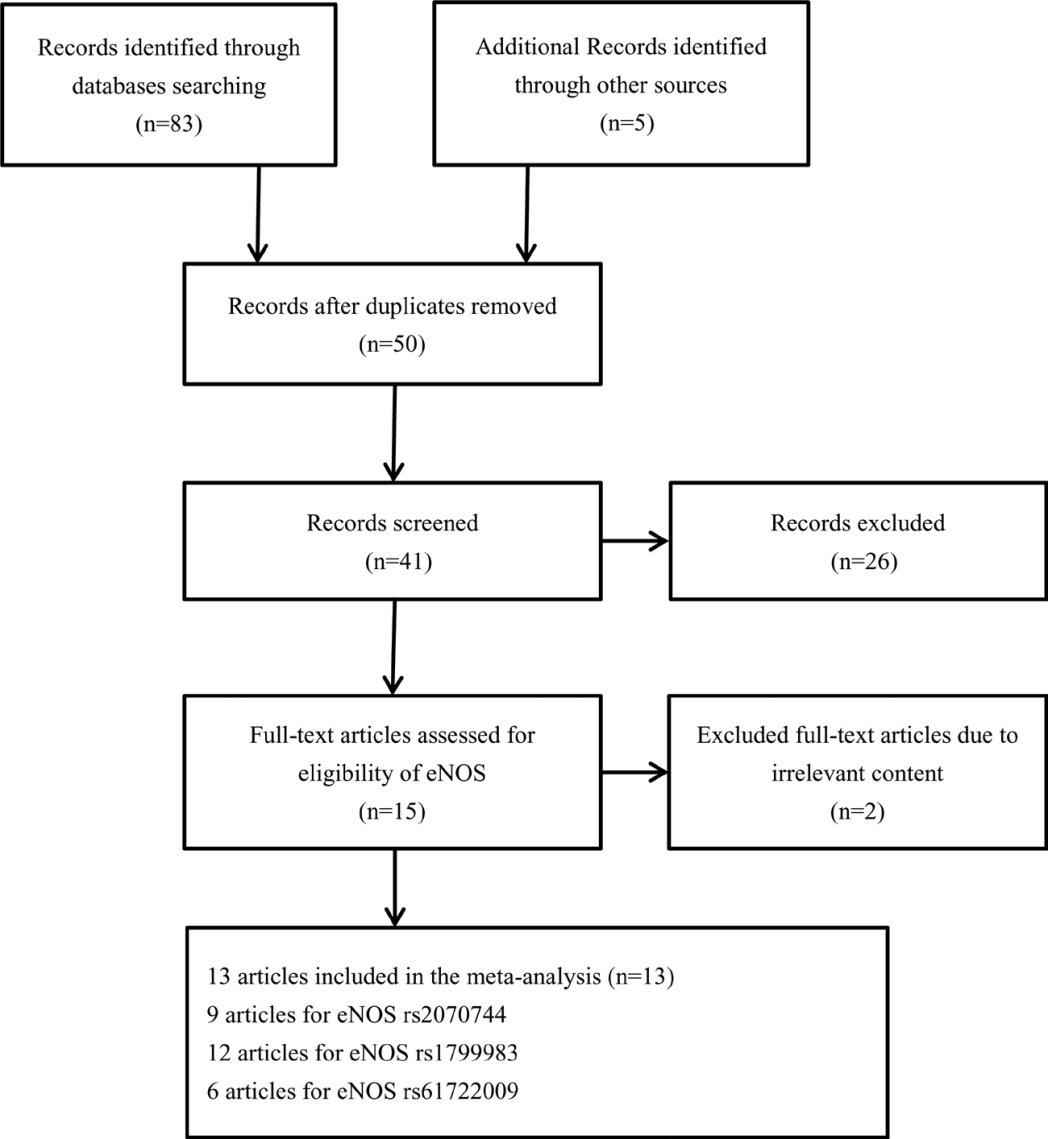
Flow chart of studies selection process for *eNOS* gene polymorphisms. eNOS = endothelial nitric oxide synthase.

The study selection process is presented in Figure 1. In total, 9 articles were included for the eNOS gene polymorphism rs2070744, with a total of 2015 cases and 1676 controls. All of the included studies were found to be in accordance with HWE. For rs1799983, 12 studies with 2780 cases and 2319 controls were included, but 3 of the studies were found to be not in compliance with HWE. For rs61722009, 6 studies with 1723 cases and 1466 controls were included, and all of the included studies had HWE *P* values > .05. Table 1 presents the characteristics of the included studies, along with the genotype frequency distributions for the 3 polymorphisms.

**Table 1:** Characteristics of eligible case-control studies included in the meta-analysis.

SNP	First author	Year	Ethnicity	Source of Control	Case			Control			HWE
					CC	CT	TT	CC	CT	TT	
rs2070744	Bianco	2013	Mixed	HB	38	93	77	25	97	79	Y
	Faure	2014	Caucasian	HB	7	27	18	3	14	17	Y
	Mostafa	2015	Caucasian	HB	66	72	82	3	36	41	Y
	Mousavi	2020	Caucasian	HB	7	47	46	7	37	56	Y
	Safarinejad	2010	Caucasian	HB	109	130	113	29	170	157	Y
	Song	2015	Asian	PB	3	46	261	2	38	268	Y
	Vucic	2017	Caucasian	PB	6	16	25	11	63	57	Y
	Ying	2013	Asian	PB	0	81	274	0	32	214	Y
	Yun	2008	Asian	PB	7	73	291	1	41	178	Y
					TT	TG	GG	TT	TG	GG	
rs1799983	Bianco	2013	Mixed	HB	110	73	25	97	93	23	Y
	Buldreghini	2010	Caucasian	HB	31	22	17	9	9	42	N
	Chavoshi	2020	Caucasian	HB	0	30	20	5	15	30	Y
	Faure	2014	Caucasian	HB	5	18	29	12	16	6	Y
	Mostafa	2015	Caucasian	HB	62	77	81	2	38	40	N
	Muti	2014	Caucasian	PB	7	10	12	8	20	20	Y
	Safarinejad	2010	Caucasian	HB	46	150	156	2	93	261	N
	Song	2015	Asian	PB	3	71	236	1	61	250	Y
	Vucic	2017	Caucasian	PB	14	60	57	12	53	66	Y
	Yan	2014	Asian	HB	14	154	410	7	127	435	Y
	Ying	2013	Asian	PB	10	96	249	4	66	176	Y
	Yun	2008	Asian	PB	1	53	371	0	25	195	Y
					4a4a	4a4b	4b4b	4a4a	4a4b	4b4b	
rs61722009	Bianco	2013	Mixed	HB	127	66	15	124	69	8	Y
	Safarinejad	2010	Caucasian	HB	45	150	157	3	92	261	Y
	Song	2015	Asian	PB	2	57	247	1	34	277	Y
	Vucic	2017	Caucasian	PB	6	33	92	3	39	89	Y
	Ying	2013	Asian	PB	7	64	284	2	27	217	Y
	Yun	2008	Asian	PB	6	72	293	2	41	177	Y

HB = hospital-based, HWE = Hardy–Weinberg equilibrium, PB = population-based, SNP = single nucleic polymorphism, Y = yes.

### 3.2. Quantitative synthesis

#### 3.2.1. rs2070744.

**Figure 2.**
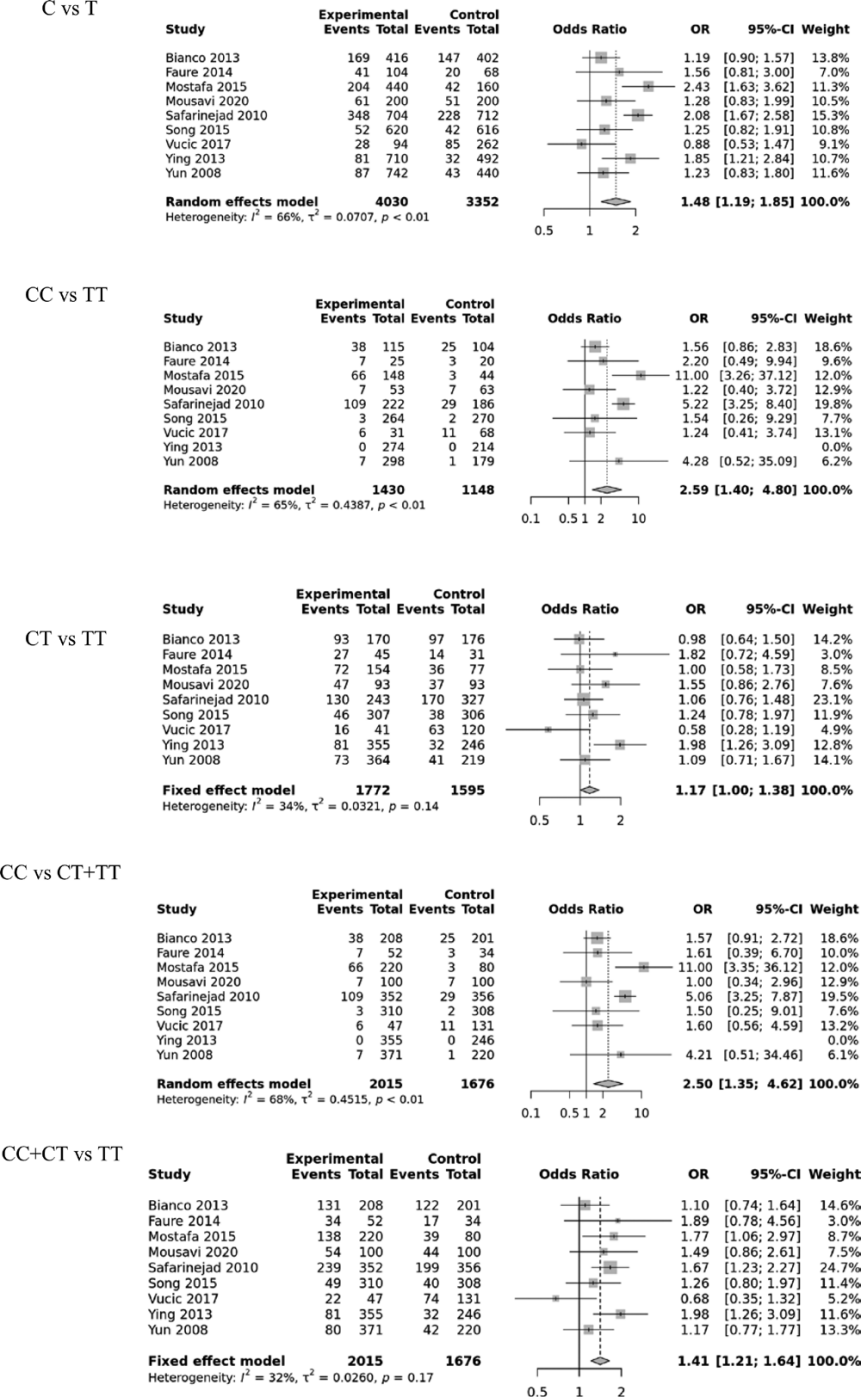
Forest plots of the eNOS rs2070744 polymorphism under different genetic models. eNOS = endothelial nitric oxide synthase.

Nine studies comprising 2015 cases and 1676 controls were included in the meta-analysis examining the association between the eNOS gene polymorphism (rs2070744) and male infertility. Results showed significant associations with male sterility risk in 5 genetic models: allele contrast C vs. T (OR, 1.48; 95% CI, [1.19–1.85]; *P* < .001), homozygote model CC vs. TT (OR, 2.59; 95% CI, [1.40–4.80]; *P* = .002), heterozygote model CT vs. TT (OR, 1.17; 95% CI, [1.00–1.38]; *P* = .0048), recessive model CC vs. TC + TT (OR, 2.50; 95% CI, [1.35–4.62]; *P* = .003), and dominant model CC + TC vs. TT (OR,1.41; 95% CI, [1.21–1.64]; *P* < .001). All studies included in the meta-analysis adhered to HWE (as shown in Fig. 2, Table 2, and Supplementary Figure S1, Supplemental Digital Content, http://links.lww.com/MD/J113).

**Table 2:** Meta-analysis of *eNOS* polymorphisms.

rs2070744 (C: Wild type, T: Mutant type)							
Model	Study	n	OR	95% CI	*P* value	*I* ^2^	*P* value (Egger test)
C vs. T	Overall	9	1.4841	[1.1905; 1.8502]	.000448	0.6625	.2686
	Asian	3	1.4014	[1.1057; 1.7763]	.00526	0.1565	.574
	Caucasian	5	1.6067	[1.1431; 2.2585]	.006342	0.7088	.2636
	Mixed	1	1.1869	[0.8953; 1.5734]	.233578	NA	NA
CC vs. TT	Overall	8	2.5902	[1.3990; 4.7955]	.002459	0.65	.48
	Asian	2	2.3711	[0.6047; 9.2982]	.215583	0	.7456
	Caucasian	5	2.9865	[1.3191; 6.7619]	.008686	0.6945	.3941
	Mixed	1	1.5595	[0.8607; 2.8256]	.14284	NA	NA
CT vs. TT	Overall	9	1.1746	[1.0012; 1.3780]	.048317	0.342	.9044
	Asian	3	1.3805	[1.0683; 1.7839]	.013686	0.4838	.782
	Caucasian	5	1.0845	[0.8590; 1.3691]	.495308	0.2901	.8719
	Mixed	1	0.9837	[0.6440; 1.5025]	.939257	NA	NA
CC vs. CT + TT	Overall	8	2.5005	[1.3530; 4.6213]	.003449	0.6808	.454
	Asian	2	2.3147	[0.5908; 9.0685]	.228353	0	.7243
	Caucasian	5	2.8322	[1.2437; 6.4493]	.013158	0.7234	.3596
	Mixed	1	1.5736	[0.9107; 2.7193]	.104233	NA	NA
CC + CT vs. TT	Overall	9	1.4120	[1.2125; 1.6444]	9.06E-06	0.3155	.6195
	Asian	3	1.4135	[1.0979; 1.8198]	.00726	0.3843	.6393
	Caucasian	5	1.5188	[1.2219; 1.8877]	.000166	0.3867	.5334
	Mixed	1	1.1017	[0.7391; 1.6421]	.634499	NA	NA
rs1799983 (T: Wild type, G: Mutant type)							
Model	Study	n	OR	95% CI	*P* value	*I* ^2^	*P* value (Egger test)
T vs. G	Overall	12	1.4069	[1.0110; 1.9577]	.042857	0.8894	.5081
	Asian	4	1.2335	[1.0531; 1.4449]	.009306	0	.4008
	Caucasian	7	1.5651	[0.8508; 2.8791]	.14978	0.9211	.1919
	Mixed	1	1.1537	[0.8614; 1.5452]	.337482	NA	NA
TT vs. GG	Overall	12	2.0848	[0.8989; 4.8355]	.086966	0.8232	.816
	Asian	4	2.0436	[1.0419; 4.0084]	.03757	0	.85
	Caucasian	7	2.2270	[0.5091; 9.7426]	.287656	0.8938	.7656
	Mixed	1	1.0433	[0.5564; 1.9563]	.894863	NA	NA
TG vs. GG	Overall	12	1.2990	[0.9515; 1.7734]	.099579	0.773	.5984
	Asian	4	1.1887	[0.9951; 1.4200]	.05671	0	.4002
	Caucasian	7	1.5074	[0.8311; 2.7342]	.176723	0.8268	.3825
	Mixed	1	0.7222	[0.3793; 1.3749]	.321757	NA	NA
TT vs. TG + GG	Overall	12	2.0007	[1.0267; 3.8990]	.04162	0.7733	.6073
	Asian	4	1.9615	[1.0019; 3.8401]	.049355	0	.7763
	Caucasian	7	2.0610	[0.5921; 7.1736]	.255763	0.8686	.9476
	Mixed	1	1.3423	[0.9150; 1.9691]	.132132	NA	NA
TT + TG vs. GG	Overall	12	1.4235	[0.9975; 2.0313]	.051637	0.8447	.5672
	Asian	4	1.2268	[1.0306; 1.4604]	.021502	0	.3628
	Caucasian	7	1.6611	[0.8687; 3.1763]	.124938	0.8753	.2311
	Mixed	1	0.8861	[0.4855; 1.6171]	.693607	NA	NA
rs61722009 (4a: Wild type, 4b: Mutant type)							
Model	Study	n	OR	95% CI	*P* value	*I* ^2^	*P* value (Egger test)
4a vs. 4b	Overall	6	1.4960	[0.9398; 2.3812]	.089448	0.8943	.2375
	Asian	3	1.5092	[1.1892; 1.9153]	.000712	0.4725	.0391
	Caucasian	2	1.8276	[0.5776; 5.7826]	.304881	0.9481	NA
	Mixed	1	0.8938	[0.6422; 1.2441]	.50571	NA	NA
4a4a vs. 4b4b	Overall	6	2.5615	[0.6999; 9.3750]	.155353	0.8047	.5956
	Asian	3	2.2155	[0.7975; 6.1550]	.127051	0	.9933
	Caucasian	2	7.1617	[0.5853; 87.6350]	.123383	0.8641	NA
	Mixed	1	0.5462	[0.2236; 1.3342]	.184456	NA	NA
4a4b vs. 4b4b	Overall	6	1.3620	[0.8691; 2.1345]	.177738	0.8073	.0562
	Asian	3	1.4978	[1.1524; 1.9468]	.002526	0.5065	.3147
	Caucasian	2	1.5212	[0.4709; 4.9141]	.483237	0.9262	NA
	Mixed	1	0.5101	[0.2029; 1.2827]	.152512	NA	NA
4a4a vs.4a4b + 4b4b	Overall	6	2.5708	[0.9055; 7.2992]	.076159	0.7683	.2023
	Asian	3	2.0926	[0.7540; 5.8079]	.156272	0	.9261
	Caucasian	2	6.1623	[0.7645; 49.6695]	.087674	0.8067	NA
	Mixed	1	0.9736	[0.6538; 1.4498]	.895288	NA	NA
4a4a + 4a4b vs. 4b4b	Overall	6	1.4435	[0.8694; 2.3966]	.15591	0.8589	.0485
	Asian	3	1.5334	[1.1870; 1.9808]	.001067	0.5028	.1839
	Caucasian	2	1.7810	[0.4818; 6.5844]	.386924	0.9453	NA
	Mixed	1	0.5333	[0.2210; 1.2870]	.161953	NA	NA

CI = confidence interval, eNOS = endothelial nitric oxide synthase, n = number, NA = not applicable, OR = odds ratio.

#### 3.2.2. rs1799983.

**Figure 3.**
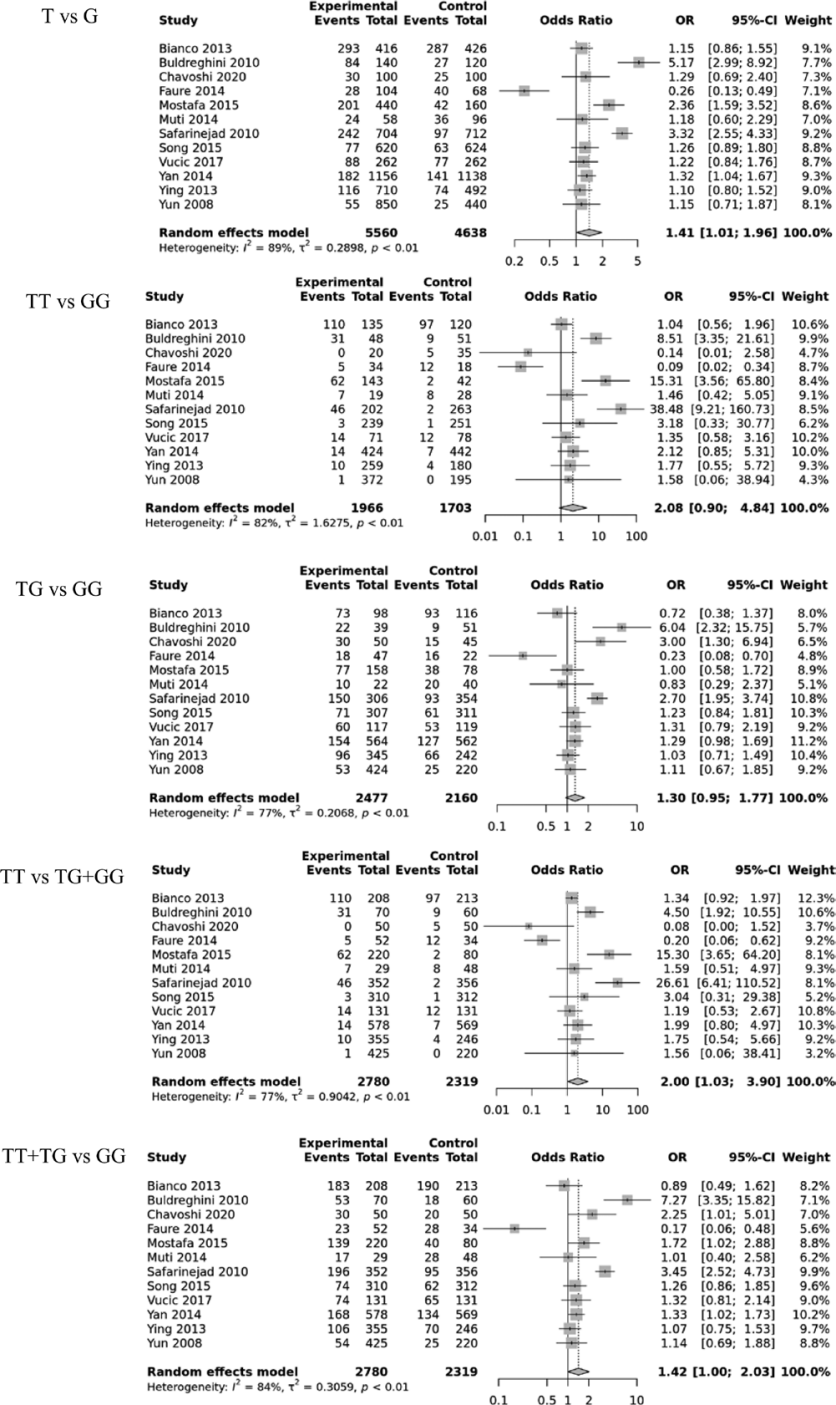
Forest plots of the eNOS rs1799983 polymorphism under different genetic models. eNOS = endothelial nitric oxide synthase.

A total of 12 randomized controlled trials (including 2780 cases and 2319 controls) were included in the meta-analysis of the rs1799983 polymorphism and its association with male infertility. The results indicated a significant correlation in 2 genetic models (allele contrast [T vs. G: OR, 1.41; 95% CI, (1.01–1.96); *P* = .043]; recessive model [TT vs. TG + GG: OR, 2.00; 95% CI, (1.03–3.90); *P* = .042]) as shown in Figure 3, Table 2 and Supplementary Figure S2, Supplemental Digital Content, http://links.lww.com/MD/J114. Subgroup analysis was conducted to investigate the effect of ethnicity, which revealed a differential correlation with Asians (allele contrast (T vs. G: OR, 1.23; 95% CI, [1.05–1.44]; *P* = .009); homozygote model (TT vs. GG: OR, 2.04; 95% CI, [1.04–4.00]; *P* = .04); recessive model (TT vs. TG + GG: OR, 1.96; 95% CI, [1.001–3.84]; *P* = .0493); dominant model (TG + TT vs. GG: OR,1.22; 95% CI, [1.03–1.46]; *P* = .021)) as shown in Supplementary Figure S4(a), Supplemental Digital Content, http://links.lww.com/MD/J115 and Table 2.

#### 3.2.3. rs61722009.

**Figure 4.**
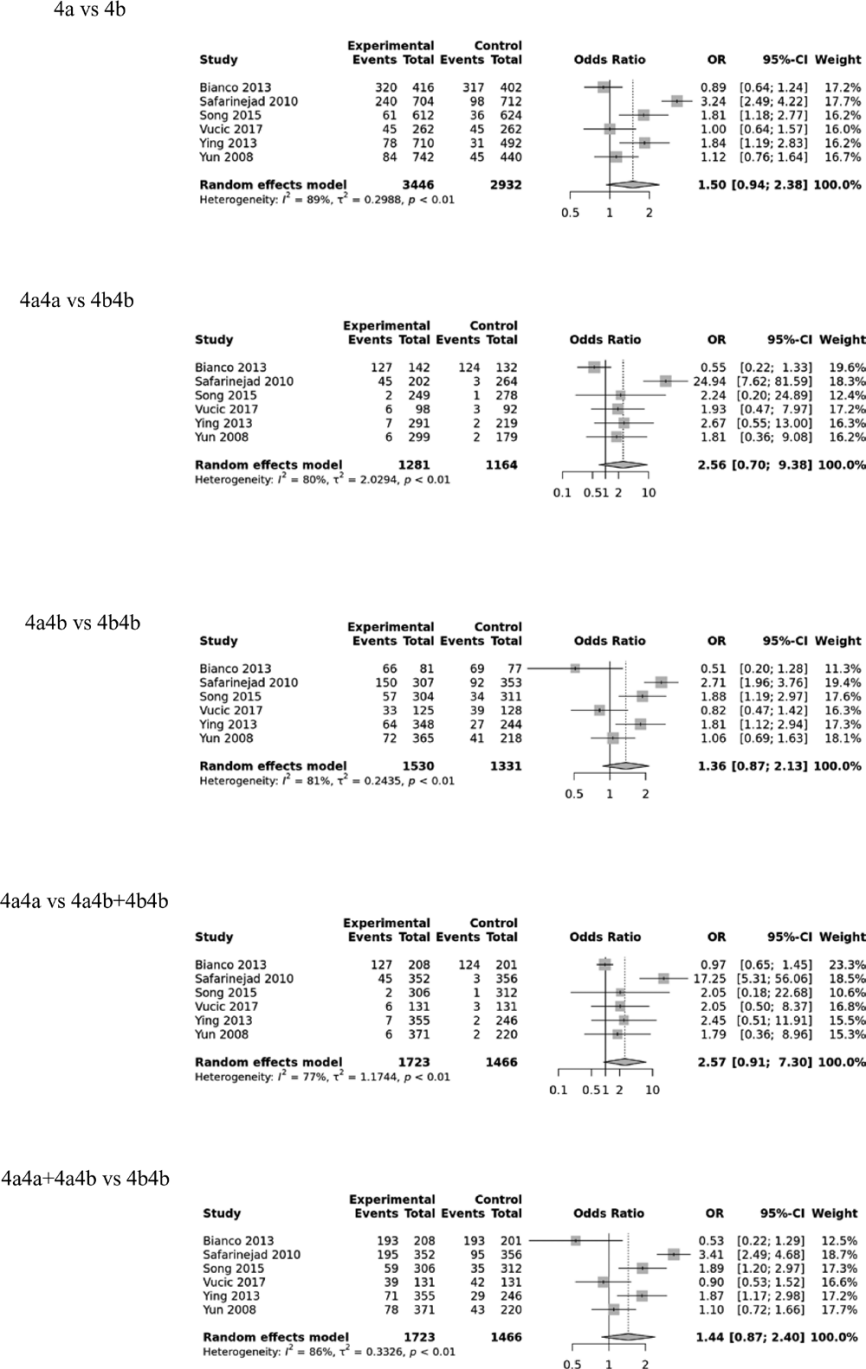
Forest plots of the eNOS rs61722009 polymorphism under different genetic models. eNOS = endothelial nitric oxide synthase.

Six studies involving 1723 infertility patients and 1466 healthy controls were included. There were no difference between the male infertility and 5 genetic models of the rs61722009 (allele contrast [4a vs. 4b: OR, 1.47; 95% CI, (.94–2.38); *P* = .09]; homozygote model [4a4a vs. 4b4b: OR, 2.56; 95% CI, (.70–9.38); *P* = .16]; heterozygote model [4a4b vs. 4b4b: OR, 1.36; 95% CI, (.87–2.13); *P* = .18]; recessive model [4a4a vs. 4a4b + 4b4b = OR, 2.57; 95% CI, (.91–7.30); *P* = .08]; dominant model [4a4a + 4a4b vs. 4b4b = OR,1.44; 95% CI, (.87–2.40); *P* = .16]). While rs61722009 had a significant difference associated with the Asians in allele contrast (OR = 1.51; 95% CI, [1.19–1.92]; *P* = .0007), the heterozygote model (OR = 1.50; 95% CI, [1.15–1.95]; *P* = .003) and dominant model (OR = 1.53; 95 CI%, [1.19–1.98]; *P* = .001) through the subgroup analysis (as shown in Fig. 4, Supplementary Figure S3, Supplemental Digital Content, http://links.lww.com/MD/J116, Supplementary Figure S4(b), Supplemental Digital Content, http://links.lww.com/MD/J115 and Table 2).

### 3.3. Sensitivity analysis and publication bias

**Figure 5.**
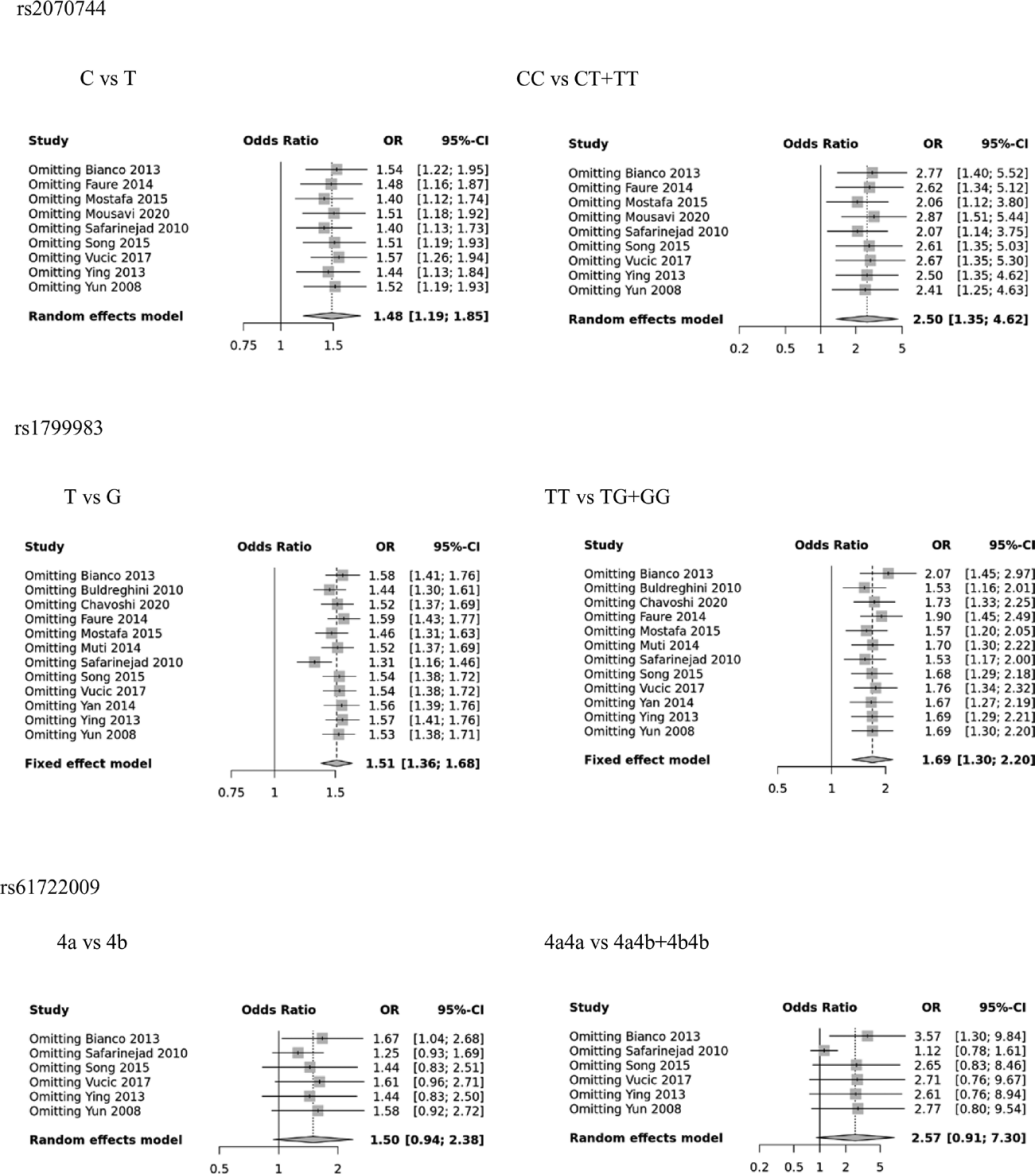
Sensitivity analysis of eNOS polymorphisms (rs2070744, rs1799983, rs61722009) in allele contrast and recessive model. eNOS = endothelial nitric oxide synthase.

A sensitivity analysis was conducted to evaluate the impact of individual studies on the overall results, and the findings indicated that the pooled ORs for the 3 polymorphisms were not significantly affected (as shown in Fig. 5). Publication bias was assessed using Egger regression tests, and the results suggested no evidence of publication bias (as displayed in Table 2 and Supplementary Figures S1–3, Supplemental Digital Content, http://links.lww.com/MD/J113; http://links.lww.com/MD/J114; http://links.lww.com/MD/J116).

### 3.4. In-silico analysis using GTEx website

**Figure 6.**
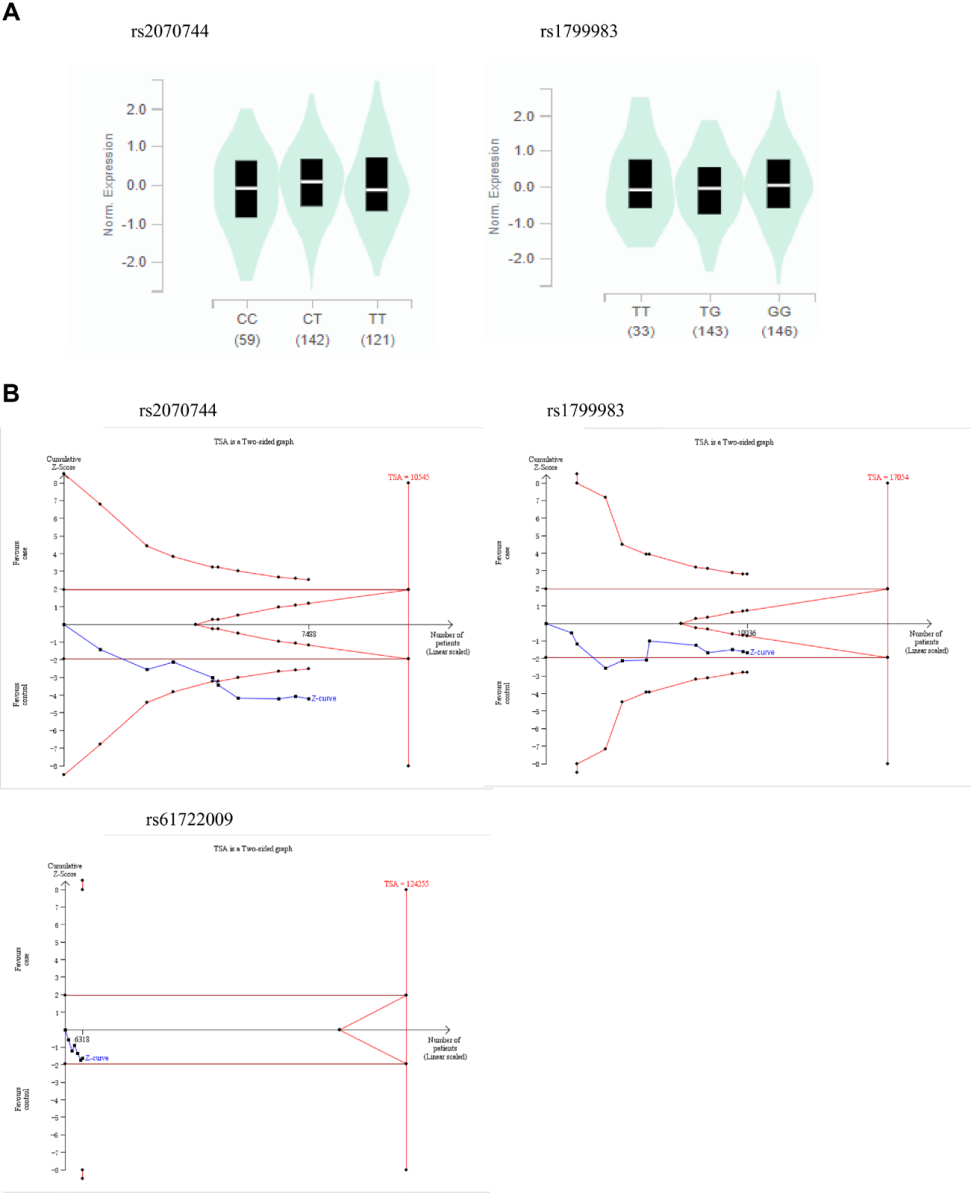
(A) eNOS mRNA expression by eQTL analysis in Human tissues based on GTEx database (rs2070744, rs1799983 in testcle). (B) TSA for eNOS 3 polymorphisms (rs2070744, rs1799983, rs61722009) under the allele contrast model. eNOS = endothelial nitric oxide synthase, TSA = trial sequential analysis.

Based on the result of the GTEx database, we observed that the polymorphism of the eNOS (rs1799983) reduce the expression of mRNA at the testicular (*P* = .036), while the rs2070744 had no difference (*P* = .16) (Fig. 6A). So far rs61722009 has not been introduced in GTEx database.

### 3.5. TSA

To assess random errors in the 3 polymorphisms, we used TSA beta 0.9. In rs2070744, the traditional and TSA boundaries are crossed by the cumulative Z-curve, indicating that even if the test sequence monitoring boundary does not reach the expected value, further tests are not required to draw a positive conclusion in advance. In rs1799983, the traditional boundary value is only crossed by the Z-curve, but the TSA boundary value is not crossed, and the test sequence monitoring boundary does not meet the expected information content, indicating that the traditional meta-analysis might have obtained false positive conclusions, and in fact, more studies need to be included to determine the conclusion. And in rs61722009, the cumulative Z-curve did not cross the traditional and TSA boundaries, indicating that further studies are required to draw a conclusion (Fig. 6B). The Preferred Reporting Items for Systematic Reviews and Meta-analysis checklist is reported in Supplementary Table S1, Supplemental Digital Content, http://links.lww.com/MD/J117.

## 4. Discussion

Mutations in genes are becoming more prevalent in many diseases.^[17]^ In recent years, many studies had found eNOS may play an important character in male infertility.^[18]^ Song et al^[19]^ have found both rs2070744 and rs61722009 as risk genotypes for male infertility in both Asian and Caucasian populations. Furthermore, no difference was observed in the eNOS gene polymorphism of rs1799983 in relation to male infertility. And Chang et al^[20]^ suggested that rs2070744 and rs6172009 of eNOS are the risk factors for developing male infertility in Asian and Caucasian populations. In addition, rs1799983 and rs2070744 may have an influence on semen quality. However, the results might be controversial due to incomplete data or obvious heterogeneity. Consequently, this updated meta-analysis was performed to demonstrate the association between genetic variations in the eNOS gene and male infertility.

This meta-analysis included a total of 13 studies with 6518 cases and 5461 controls and aimed to investigate the association between 3 polymorphisms (rs2070744, rs1799983, and rs61722009) and the risk of male infertility. The results showed that rs2070744 was significantly associated with the risk of male infertility in 5 genetic models among mixed, Asian, and Caucasian populations. Additionally, rs61722009 was associated with the risk of male infertility in Asians. Subgroup analysis revealed differences in rs1799983 related to male infertility. These findings suggest that genetic variants in eNOS may play a role in the susceptibility to male infertility. However, further studies are needed to better understand the underlying mechanisms involved.

Previous studies have suggested that the eNOS rs2070744 polymorphism is a risk factor for male infertility in both Asian and Caucasian populations. Our meta-analysis confirmed this association and showed significant heterogeneity among the 5 genetic models, which may be due to differences in ethnicity. Therefore, we conducted a subgroup meta-analysis by ethnicity and found that rs2070744 was a risk factor for male infertility in the allelic contrast, heterozygote model, and dominant model among Asian populations. Additionally, TSA analysis indicated that further case-controls are not necessary to confirm this result.

In contrast to previous studies, our meta-analysis found that eNOS rs1799983 was significantly related to male infertility in 2 genetic models. Interestingly, subgroup meta-analysis stratified by ethnicity also showed consistent results. However, TSA analysis and the GTEx database suggested that the traditional meta-analysis might have obtained false positive conclusions and that rs1799983 actually reduces the expression of mRNA in the testes. Therefore, further studies are needed to confirm the association between rs1799983 and male infertility.

Regarding eNOS rs61722009, few studies reported its relationship with the risk of male infertility, especially after excluding studies with high heterogeneity and including new studies with lower heterogeneity. We have found the associations only with the Asian populations. Combines with the small sample size nevertheless, the findings from the current analysis are inconclusive according to the results of the TSA.

The eNOS gene expression can be regulated by transcription, translation, protein phosphorylation, and other aspects, which have been confirmed by previous studies.^[21]^ Hypoxia and strenuous exercise can upregulate eNOS expression levels. In our research, rs2070744 can raise DNA susceptibility in all 5 genetic models because the T786C variant is a cytosine (C) replacement of the thymine nucleotide (T) at the 786 loci of the eNOS.^[22]^ This leads to structural changes in the protein that alter the activity of eNOS. The increasing expression of eNOS leads to elevated production of NO, which directly affects the function and viability of sperm.^[23]^

In the research, we performed a systematic and comprehensive search to obtain reliable results. We used sensitivity analysis, Egger tests to evaluate the quality of the included studies. Nonetheless, there are some limitations in this meta-analysis. First, only 3 ethnic groups (Asian, Caucasian, and Mixed) were included in this study. Second, the sample size of rs1799983 and rs61722009 was relatively small. Third, the language of including studies was only English, which may influence the association between them. Fourth, the number of case-controls was too small which might result in great errors.^[24,25]^ More large sample size case-control studies of various ethnicities are needed to investigate the functions of eNOS polymorphisms.

## 5. Conclusion

The results of this meta-analysis suggest that eNOS rs2070744 and rs1799983 are associated with the risk of male infertility, and eNOS rs61722009 is associated with the risk of male infertility in Asian population. However, further well-designed case-control studies are needed to confirm these findings.

## Author contributions

**Data curation:** Zhihai Teng, Hu Wang, Fengran Guo, Zhenwei Han, Yaxuan Wang.

**Formal analysis:** Zhihai Teng, Hu Wang, Zhenwei Han, Yaxuan Wang.

**Funding acquisition:** Zhenwei Han.

**Methodology:** Fengran Guo, Zhenwei Han.

**Project administration:** Zhihai Teng, Zhenwei Han.

**Software:** Zhihai Teng, Hu Wang, Fengran Guo, Yaxuan Wang.

**Writing – original draft:** Zhihai Teng, Hu Wang.

**Writing – review & editing:** Zhihai Teng, Hu Wang, Zhenwei Han, Yaxuan Wang.

## Supplementary Material
