# Supplementary figures and images for: eNOS polymorphisms on male infertility: An updated systematic review and meta-analysis
Source: Medicine (Baltimore). 2023 Jun 16;102(24):e33993. doi: 10.1097/MD.0000000000033993 (PMC10270503; doi:10.1097/MD.0000000000033993)

Supplementary Figure 1. Funnel plot of eNOS rs2070744 polymorphism under different genetic models.

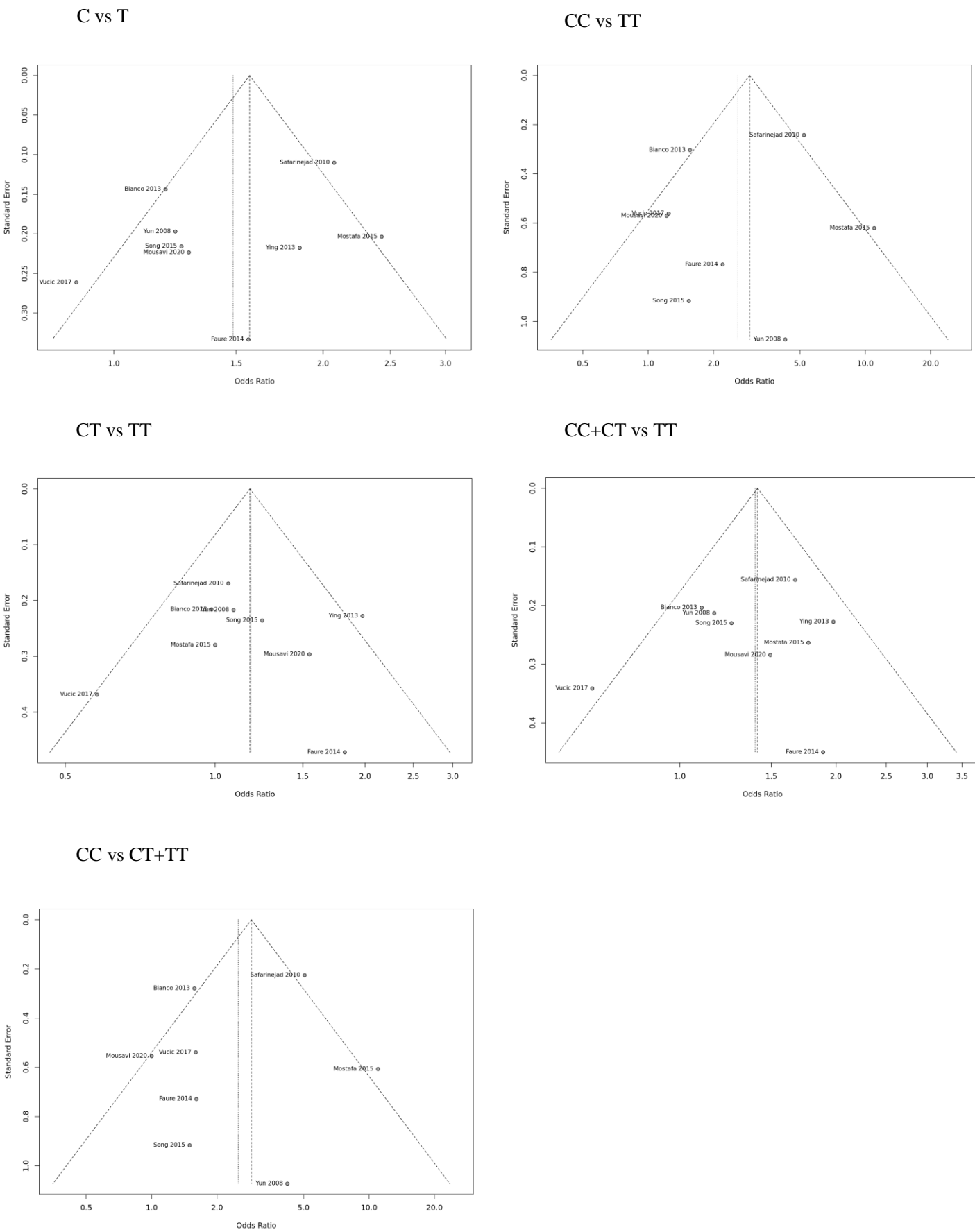

Supplement: Supplementary file 1 [file medi-102-e33993-s001.pdf]
