# Supplementary material for: eNOS polymorphisms on male infertility: An updated systematic review and meta-analysis
Source: Medicine (Baltimore). 2023 Jun 16;102(24):e33993. doi: 10.1097/MD.0000000000033993 (PMC10270503; doi:10.1097/MD.0000000000033993)

Supplementary Figure 2. Funnel plot of eNOS rs1799983 polymorphism under different genetic models.

T vs G

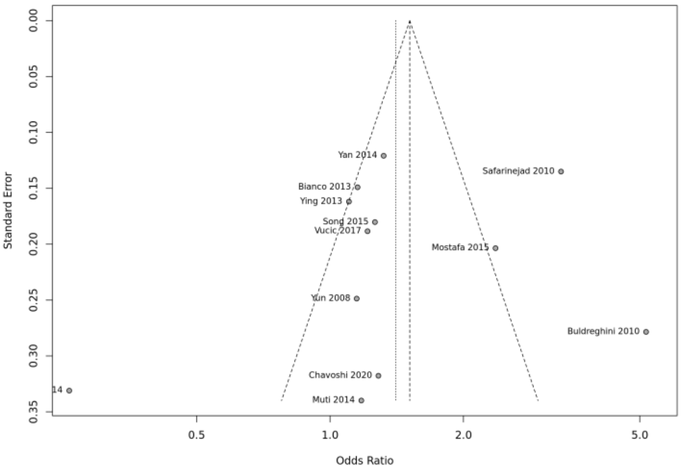

TT vs GG

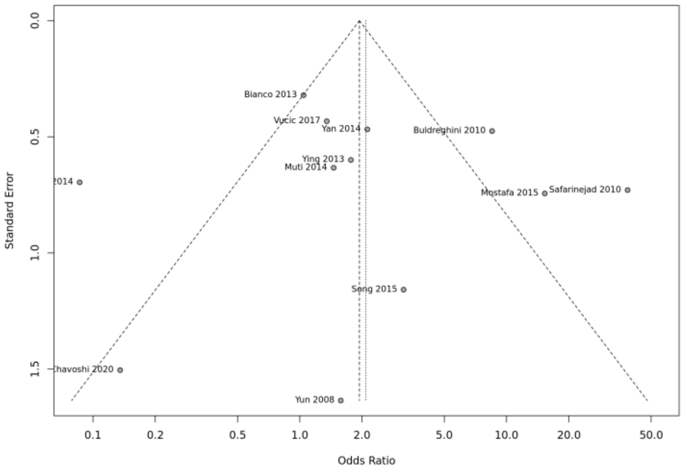

TG vs GG

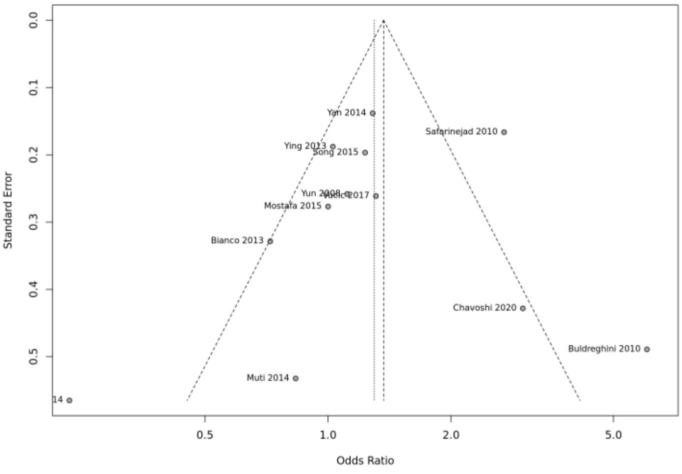

TT+TG vs GG

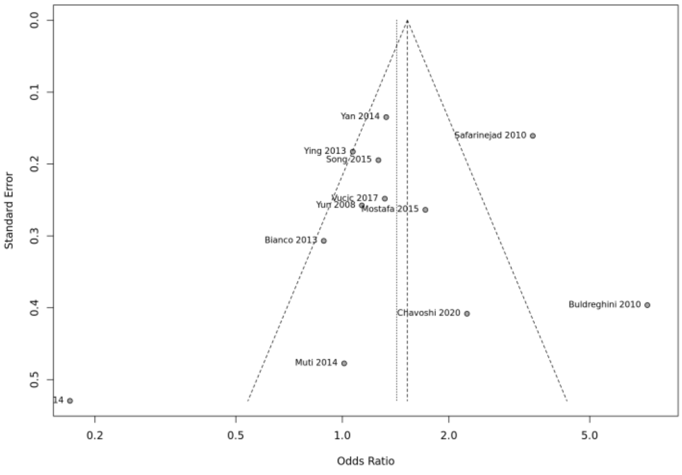

TT vs TG+GG

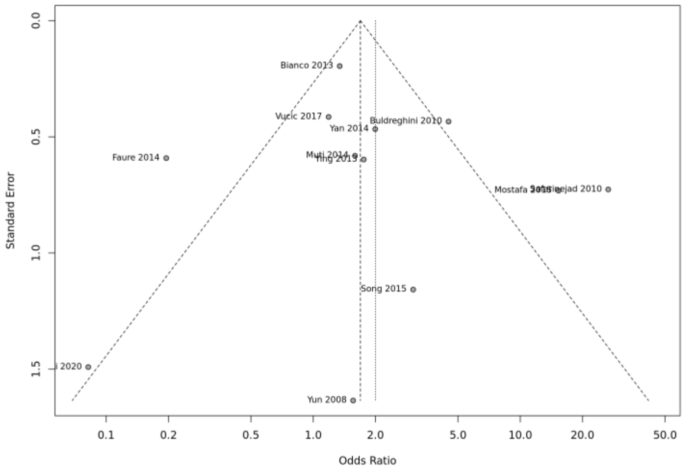

Supplement: Supplementary file 2 [file medi-102-e33993-s002.pdf]
