# Supplementary material for: eNOS polymorphisms on male infertility: An updated systematic review and meta-analysis
Source: Medicine (Baltimore). 2023 Jun 16;102(24):e33993. doi: 10.1097/MD.0000000000033993 (PMC10270503; doi:10.1097/MD.0000000000033993)

Supplementary Figure 4. Subgroup analysis of eNOS rs1799983 and rs617722009 polymorphism under allele contrast model.

T vs G

(a)

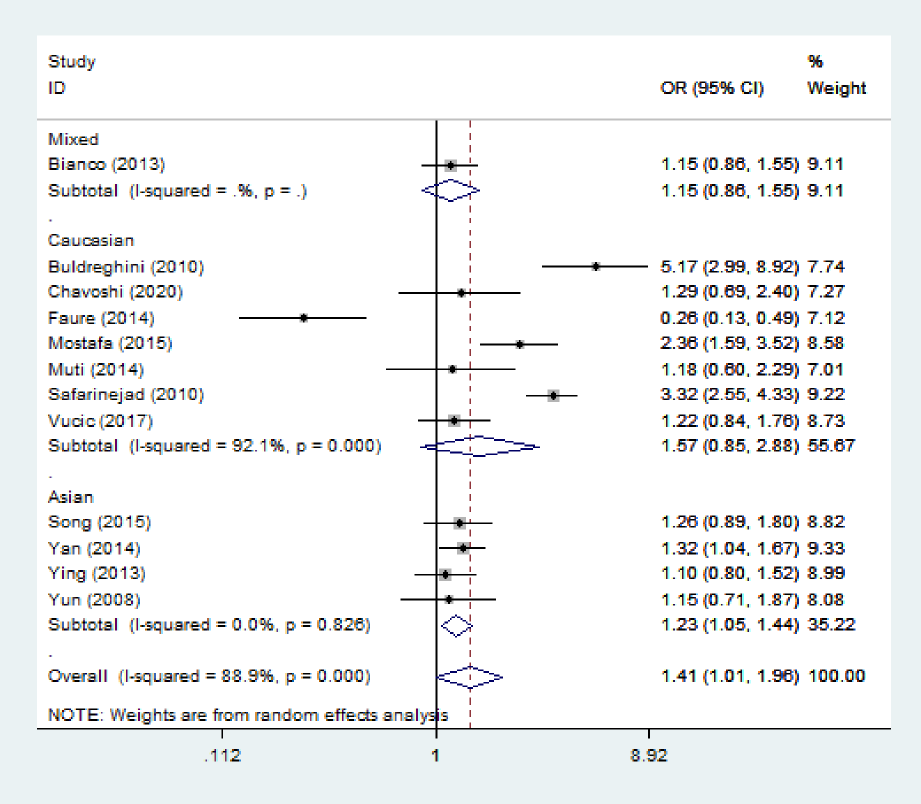

4a vs 4b

(b)

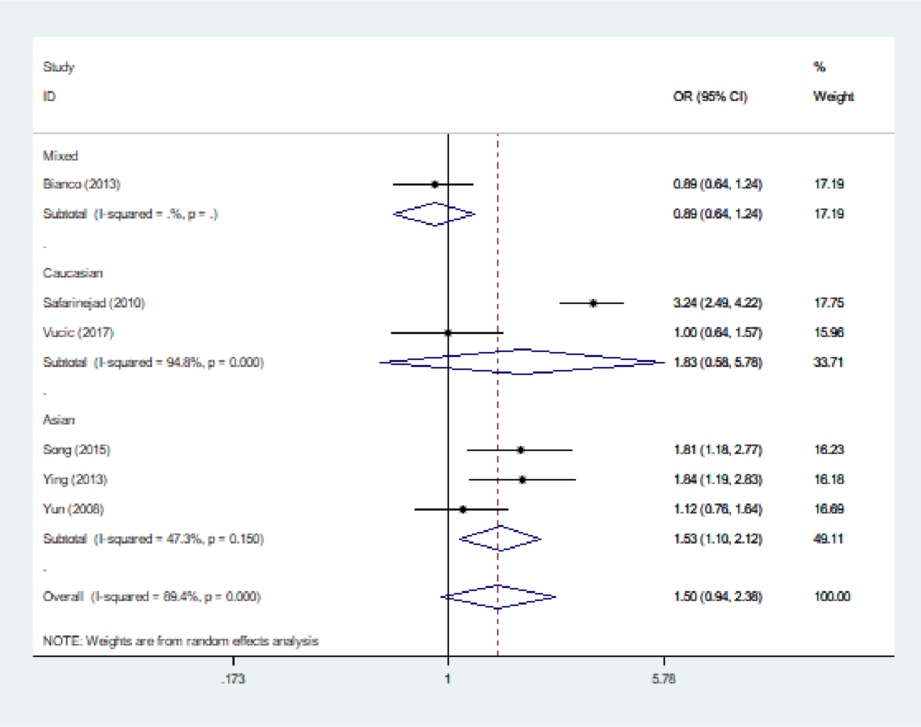

Supplement: Supplementary file 3 [file medi-102-e33993-s003.pdf]
