# Supplementary material for: eNOS polymorphisms on male infertility: An updated systematic review and meta-analysis
Source: Medicine (Baltimore). 2023 Jun 16;102(24):e33993. doi: 10.1097/MD.0000000000033993 (PMC10270503; doi:10.1097/MD.0000000000033993)

Supplementary Figure 3. Funnel plot of eNOS rs617722009 polymorphism under different genetic models.

4a vs 4b

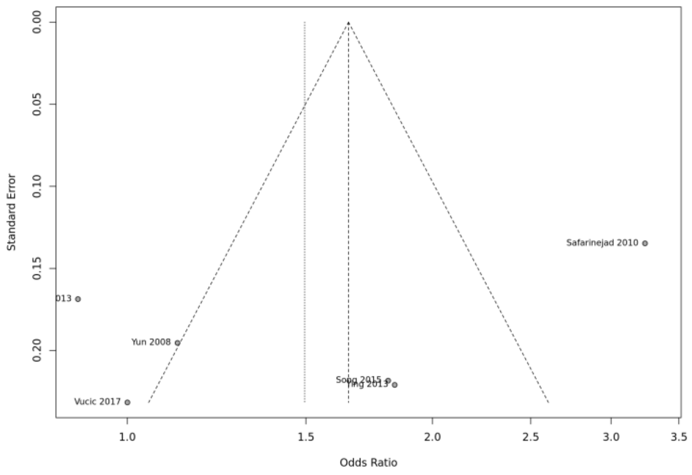

4a4a vs 4b4b

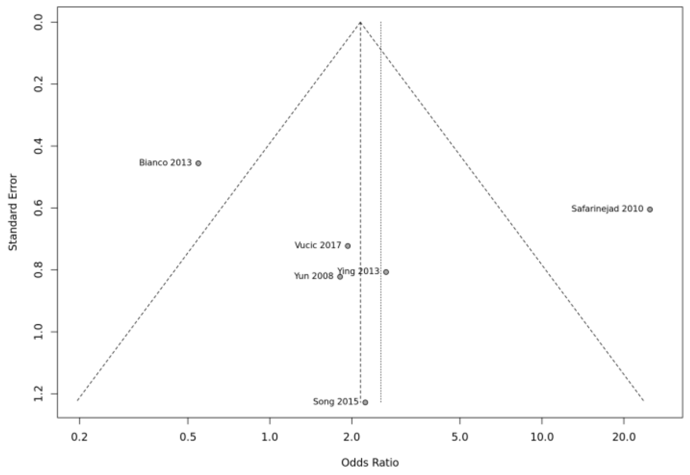

4a4b vs 4b4b

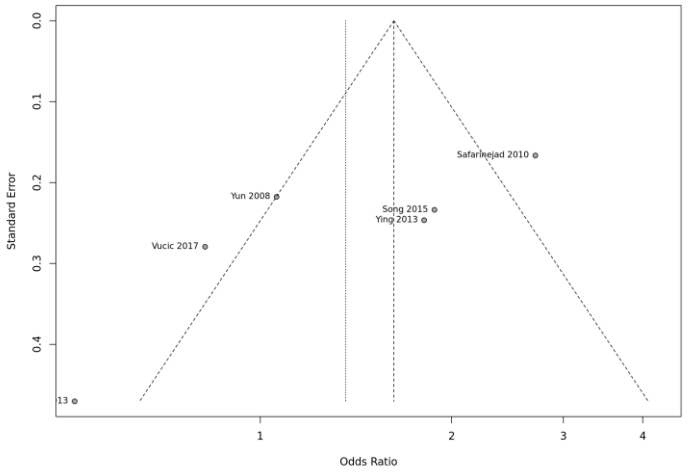

4a4a+4a4b vs 4b4b

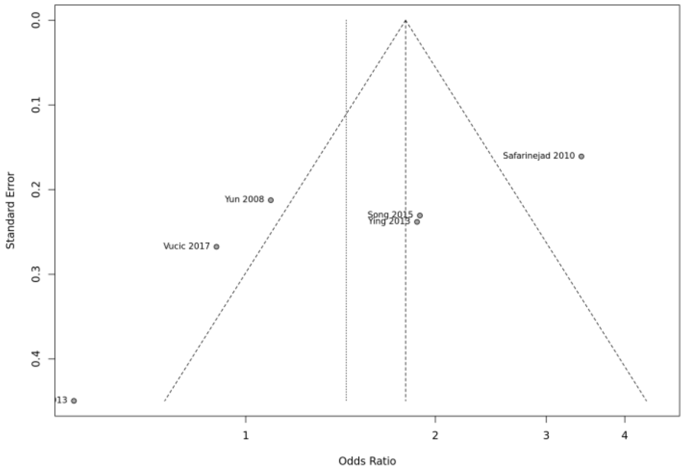

4a4a vs 4a4b+4b4b

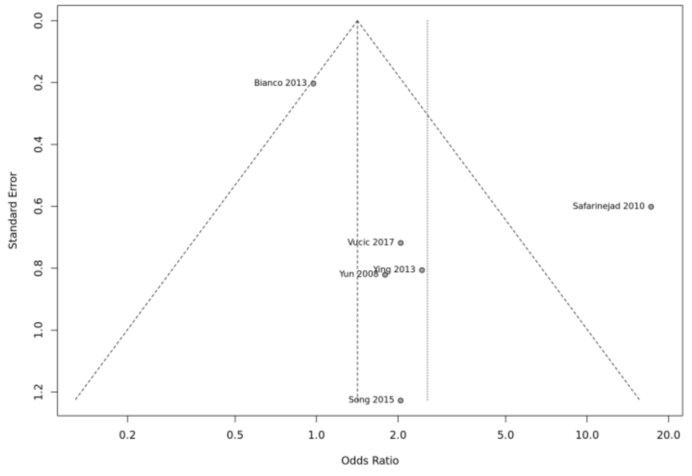

Supplement: Supplementary file 4 [file medi-102-e33993-s004.pdf]
